# Fatalities in opioid agonist treatment with long-acting injectable buprenorphine

**DOI:** 10.1007/s00414-025-03535-w

**Published:** 2025-06-18

**Authors:** Margareeta Häkkinen, Claudia Mariottini, Pirkko Kriikku, Ilkka Ojanperä

**Affiliations:** 1 Forensic Toxicology Unit, Finnish Institute for Health and Welfare, P.O. Box 30, Helsinki, 00271 Finland; 2 Department of Psychiatry, University of Turku, Turku, Finland; 3 Department of Forensic Medicine, University of Helsinki, Helsinki, Finland

**Keywords:** Buprenorphine-related death, Buprenorphine depot, Opioid use disorder, Poisoning, Prolonged-release buprenorphine

## Abstract

**Purpose:**

Long-acting injectable buprenorphine (LAI BUP) is a novel opioid agonist treatment (OAT) medication with several benefits for patients, OAT units, and society. We present a case series of fatalities in OAT with LAI BUP.

**Methods:**

This was an audit of OAT patient records, supplemented with results of post-mortem (PM) investigation. We started with all deaths (*N* = 604) between 2018 and 2020 in Finland with a buprenorphine or norbuprenorphine finding in PM toxicology and with known substance use history or concomitant findings of illicit drugs. We then examined the patient records of the 43 individuals in OAT at the time of death. We analyzed information on OAT medication, OAT structure, and performance, including concomitant substance use, and PM findings.

**Results:**

Ten LAI BUP patients died during the study period, all of them in 2020, out of the 18 patients receiving some OAT that year. Among the LAI BUP patients, four died from buprenorphine poisoning. Eight patients had PM findings of abused drugs, always including unprescribed benzodiazepines. Two patients showed signs of additional buprenorphine use of which their OAT unit was unaware. Three patients were classified as non-compliant with OAT but had received no extra support to tackle their situation. Visits to the OAT clinic had mainly occurred only on injection days.

**Conclusions:**

LAI BUP comprised 56% of OAT medication among all deceased OAT patients in 2020. Despite its recognized benefits, LAI BUP treatment was associated with deaths. Psychosocial support among the deceased LAI BUP patients seemed inadequate.

## Introduction

Long-acting injectable buprenorphine (LAI BUP) became available in Finland in January 2019. There is no register or database of patients receiving opioid agonist treatment (OAT) in Finland, but the estimated number of OAT patients in 2019 was 4729 [1]. At the end of 2019, the most common OAT medication, with a 52% share, was sublingual buprenorphine-naloxone (BUP-NAL), whereas the proportion of LAI BUP was 12%, and methadone 35% [1]. Of the patients enrolled in LAI BUP OAT, 61% received weekly injections and 39% monthly [1]. LAI BUP has since become more popular in Finland, but the exact proportions of OAT medications after the end of 2019 are unavailable. According to an unpublished estimation by OAT medication companies based on sales reports, the number of LAI BUP patients more than doubled from 2019 to 2020. In Finland, OAT is free of charge regardless of the medication, when delivered from an OAT unit. The choice of OAT medication is individual between BUP-NAL, LAI BUP, and methadone, and a doctor chooses the medication based on a patient’s medical condition and preference.

Weekly and monthly subcutaneous LAI BUP formulations have proved to be efficient in treating opioid use disorder when compared to sublingual BUP-NAL formulations [2]. LAI BUP may be beneficial for patients at the initiation of OAT, when discharged from prison or hospital, when avoiding diversion or misuse of buprenorphine or methadone, or when compliant patients wish to avoid daily oral dosing [3, 4]. Public health benefits may derive from increased OAT retention rates, reduced diversion risk, and reduced need for healthcare resources [4, 5]. In a systematic review, LAI BUP was positively associated with improvements in social determinants of health, such as abstinence, accessibility to opioid use disorder health programs, employment, social relationships, and crime reduction [6].

OAT is efficient in reducing the mortality of people with opioid use disorder [7]. However, as the most abused opioid in Finland, buprenorphine is also associated with a high number of deaths every year. Most Finnish opioid users started and have continued their opioid abuse with buprenorphine, which is different from, e.g., other Nordic countries [8]. The early introduction of buprenorphine in the 1990s by two general practitioners with questionable OAT methods likely promoted the common parenteral use of buprenorphine [9]. The majority of the buprenorphine abused in this country is mono-buprenorphine smuggled from abroad, but BUP-NAL diverted from OAT is found to a lesser extent in buprenorphine deaths [10, 11]. Buprenorphine-related deaths mostly occur outside OAT. We have previously shown that among buprenorphine-related deaths during 2018–2020 in Finland, the proportion of individuals receiving OAT within a year before death was only 10% [9]. To the best of our knowledge, no published data on LAI BUP fatalities exist in the scientific literature.

In this population-based study in Finland during 2018–2020, our objective was to investigate the role of LAI BUP in the fatalities of OAT patients. We report for the first time the OAT characteristics and post-mortem (PM) toxicological findings for the deceased who had received LAI BUP.

## Materials and methods

### Medico-legal cause-of-death data

A medico-legal cause-of-death investigation in Finland covers all sudden and unexpected deaths that may be related to poisoning, accident, crime, suicide, occupational disease, medical procedure, or war. Approximately 16% of all deaths in Finland undergo a medico-legal investigation, and a comprehensive PM toxicology investigation is conducted in 75% of autopsy cases (12% of all deaths).

Our medico-legal data consisted of toxicological laboratory analysis results extracted from the national PM toxicology database [12], and information extracted from death certificates: time of death, age, sex, cause of death, manner of death, main autopsy findings, and a brief description of the circumstances of death. PM drug concentrations were measured from femoral blood. We excluded hospital-administered drugs from the PM toxicology data.

The PM toxicological investigation has been described in detail elsewhere [9]. The concentrations of buprenorphine (BUP) and norbuprenorphine (NBUP) in PM blood refer to non-conjugated species, without hydrolysis prior to analysis. The lower limit of quantification (LLOQ) for buprenorphine and norbuprenorphine in blood was 0.2 µg/L.

### Data collection and interpretation

This study material originates from our previous study [9] in which we investigated all deaths (*N* = 604) in Finland in 2018–2020 with a BUP or NBUP finding in PM toxicology and with a known substance use history in a referral to the toxicology unit or in the death certificate, or concomitant findings of illegal drugs in PM toxicology. We requested all substance use service units offering OAT to send us information on whether the individuals had received OAT within 12 months before death. If in OAT, we asked the OAT units to provide patient records with information on OAT treatment plan, duration, medication, prescribed medications, and OAT performance, including concomitant substance use. These data were consistently reported for all cases, although the accuracy of the information depended on how thoroughly the personnel at the OAT units had recorded it.

**Fig. 1:**
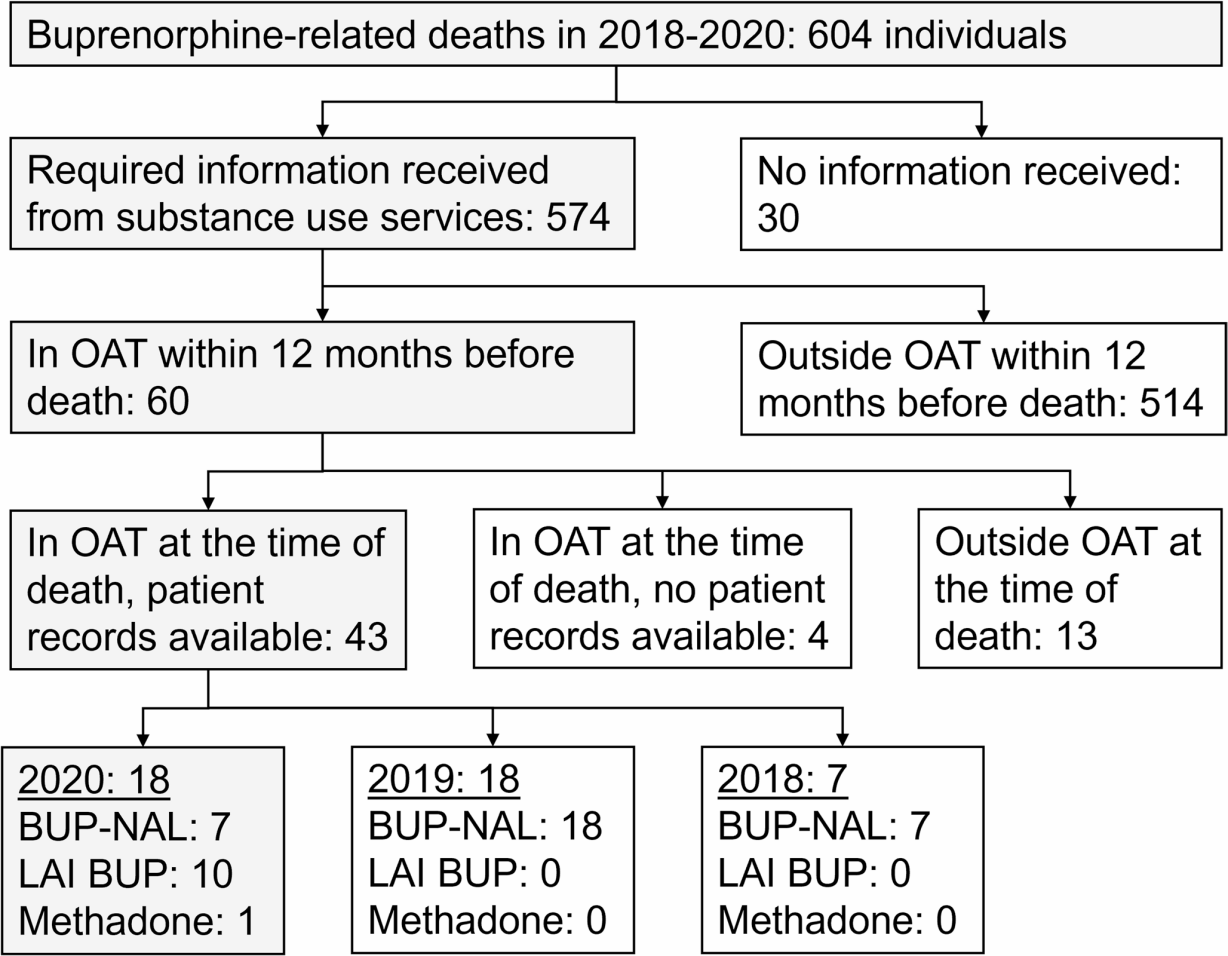
Flowchart of the study case selection. OAT opioid agonist treatment, BUP-NAL buprenorphine-naloxone, LAI BUP long-acting injectable buprenorphine

In the current study, we examined a subgroup of the ten deceased who had received LAI BUP as their OAT medication (Fig. 1). All the studied LAI BUP patients had died in 2020.

We investigated all the patient records of the ten deceased LAI BUP patients to gather information concerning their OAT performance. We collected information on concomitant substance use and the status of present intravenous substance use from the patient records. In the patient records, these data were based on urine drug screens and patients’ self-reported use.

We classified an individual as non-compliant with OAT in cases where the treatment had not been conducted as planned within three months before the death. This included, for example, intoxications, missed clinic visits, and other missed contacts with OAT personnel. According to this evaluation, a patient may have been compliant with OAT even when concomitant substance use was present, as long as the concomitant use did not result in severe intoxications or other complications or missed contacts with the OAT unit.

### Ethics approvals

We carried out the study based on research permit THL/2509/6.02.00/2021, issued by the Finnish Institute for Health and Welfare.

## Results

### LAI BUP cases and treatment practices

LAI BUP comprised 56% of OAT medication among all deceased OAT patients in 2020, with no cases in 2018–2019 (Fig. 1). As illustrated in Fig. 1, of the studied death cases, in 18 the deceased had been enrolled in OAT, and of them, 10 had received LAI BUP. Table 1 provides basic demographic and clinical data of the deceased LAI BUP patients.

**Table 1:** Sex, age, diseases and concerns, and prescriptions of deceased patients who received long-acting injectable buprenorphine (LAI BUP) treatment

Patient	Sex	Age	Documented diseases and concerns	Prescriptions
1	Male	25	HCV	Glecaprevir-pibrentasvir 100/400 mg 3 × 1
2	Male	25	HCV, psychotic symptoms, intoxications, suicide attempts	Olanzapine 300 mg i.m./2 weeks, quetiapine 200 mg 1 × 1
3	Male	50	HCV, epilepsy, anxiety	Oxazepam 15 mg, 4 tablets/week
4	Female	42	HCV, agoraphobia with panic disorder	None
5	Male	24	Asthma	Oxazepam 15 mg 1 × 3, zopiclone 7,5 mg 1 × 1, quetiapine 25 mg 1 × 1, cetirizine 10 mg 1 × 1, budesonide 200 µg 1-2 × 1-2, salbutamol 100 µg 1-2 × 1-4
6	Female	32	Borderline personality disorder, mixed anxiety and depressive disorder	Vortioxetine, melatonin, olanzapine (doses unknown)
7	Male	38	Unknown	Quetiapine 25 mg 0.5 × 1, melatonin 3 mg 1-2 × 1, testosterone 50 mg 1 × 1 transdermal
8	Male	41	HCV, ADHD	Oxazepam 30 mg 1 × 3
9	Male	46	HCV, diabetes, asthma	Insulin
10	Male	25	HCV, generalized anxiety disorder	Oxazepam 15 mg 1 × 3, vortioxetine 20 mg 1 × 1, gabapentin 300 mg 1 × 3, mirtazapine 15 mg 1 × 1, quetiapine 25 mg 2 × 1, melatonin 3 mg 1-2 × 1

HCV hepatitis C virus, ADHD attention deficit hyperactivity disorder, i.m. intramuscular

All the LAI BUP patients were in OAT for the first time. One patient had started OAT with LAI BUP, and three patients had started their OAT with a two-week sublingual induction period and switched to LAI BUP after that. All the patients had started LAI BUP treatment voluntarily, and none had started LAI BUP because of buprenorphine diversion. One patient had previously received weekly LAI BUP for 2.5 months but had been transferred to sublingual BUP-NAL two weeks prior to death because of acute treatment at a psychiatric ward.

Table 2 lists OAT medication and characteristics. Visits to OAT units usually occurred only when patients received their buprenorphine injections. As an example, before OAT initiation, patient #2 visited a nurse in a substance abuse unit for psychosocial support 1–2 times a month. After initiating OAT, he only visited the clinic on injection days to receive LAI BUP but did not have any nurse appointments for psychosocial treatment and support. In addition, patient #2 had psychotic symptoms and complications related to intravenous drug abuse after OAT initiation, and OAT did not improve his health. The patient records showed no indication that the COVID-19 pandemic would have reduced psychosocial treatment among the studied individuals.

**Table 2:** Medications and OAT characteristics for LAI BUP patients

Patient	LAI BUP dose, mg	OAT duration	LAI BUP duration	Days since last LAI BUP	Visits to OAT unit during LAI BUP	Compliant/non-compliant OAT	Known concomitant substance use	Known iv use
Weekly injections								
1	16	2 y 8 m 2 w	4 m	1	Only on injection days	Compliant	BZD, THC	No
2	16	4 m 2 w	4 m	1	2–5 phone calls/month, 1 visit to a nurse, otherwise visits only on injection days	Non-compliant: missed visits to the clinic, and prolonged, unplanned injection intervals	BZD, PRG, AMP, MDMA, tramadol	Yes
3	24	3 m 1 w	3 m 1 w	3	Only on injection days	Compliant	BZD, THC	No
4	32	8 y 4 m	6 m 3 w	6	Only on injection days	Compliant	BZD daily, AMP monthly	Yes
5	32	3 m 3 w	3 m 1 w	2	Nurse’s appointment and psychosocial treatment but only on injection days	Compliant	THC, BZD, PRG	Yes
Monthly injections								
6	64	5 m 3 w	4 m 1 w	23	Only on injection days	Compliant	Occasional EtOH	No
7	128	> 11 y	7 m	5	Few phone calls, one extra visit to a nurse, otherwise only on injection days	Compliant	AMP, THC, BZD, PRG	Yes
8	128	1 y 5 m	5 m 2 w	12	1–2 visits to a nurse/month, 2 visits to a doctor during LAI BUP treatment	Non-compliant: missed visits to the clinic and missed calls from the clinic	AMP, THC	No
9	128	2 y 7 m	4 m 2 w	22	Unknown	Non-compliant: sounded intoxicated on the phone	Occasional THC, BZD	No
10	128	2 y 3 m	3 m 2 w	20	Only on injection days	Compliant	BZD, THC, previously AMP	Yes

OAT opioid agonist treatment, LAI BUP long-acting injectable buprenorphine, y year, m month, w week, iv intravenous, AMP amphetamine, BUP buprenorphine, BZD benzodiazepines, EtOH ethanol, MDMA 3,4-methyl​enedioxy​methamphetamine (ecstasy), PRG pregabalin, THC tetrahydrocannabinol

### Post-mortem toxicology and concomitant substance use

The PM toxicology results are listed in Table 3. Four patients died from buprenorphine poisoning as determined by the forensic pathologist.

**Table 3:** Findings of post-mortem investigation for LAI BUP patients. Concentrations were measured in post-mortem femoral blood

Patient	Cause of death	Manner of death	BUP and NBUP concentrations, µg/l	Other post-mortem findings, mg/l	Additional findings besides known substance use ^a)^
1	Poisoning (BUP, CLO, PRG, DZP, AMP)	Accident	BUP 1.2, NBUP 2.6, BUP/NBUP 0.46	7-amino-CLO 0.35, NDZP < 0.02, AMP 0.91, THC-COOH 0.0035, PRG 10	AMP, PRG
2	Hanging	Suicide	BUP 15, NBUP 9, BUP/NBUP 1.7	Olanzapine* 0.10, norolanzapine* 0.074, lithium 2.8	Additional lithium use (previously attempted suicide with lithium)
3	Longterm substance use	Disease	BUP 3.9, NBUP 5.8 BUP/NBUP 0.67	Alprazolam 0.042, hydroxyalprazolam 0.001, 7-amino-CLO 0.029, oxazepam* 0.031, NDZP < 0.02, THC-COOH 0.0073	
4	Poisoning (BUP, AMP, DZP)	Accident	BUP 32, NBUP 8.9, BUP/NBUP 3.6	EtOH 0.80‰, DZP 0.036, NDZP 0.054, AMP 0.56	EtOH
5	Anafylactic shock after AMP iv use	Accident	BUP 12, NBUP 11, BUP/NBUP 1.1	CLO < 0.01, 7-amino-CLO 0.21, AMP 0.44, THC 0.0018, THC-COOH 0.06	AMP
6	Acute liver failure	Unknown	BUP 2.1, NBUP 1.7, BUP/NBUP 1.2	Vortioxetine* 0.041	No substance use detected
7	Poisoning (BUP, PRG, BZD, AMP)	Accident	BUP 5.4, NBUP 4.1, BUP/NBUP 1.3	Oxazepam 0.042, DZP 0.041, NDZP 0.022, AMP 1.9, THC 0.0086, THC-COOH 0.23, 11-OH-THC 5, PRG 19, gabapentin* <1.0	
8	Stabbed in the neck	Homicide	BUP 7, NBUP 6.5, BUP/NBUP 1.1	CLO 0.005, 7-amino-CLO 0.026, oxazepam* 0.067, AMP 1.6, THC 0.0021, THC-COOH 0.057	BZD
9	Poisoning (BUP)	Unknown	BUP 21, NBUP 6.7, BUP/NBUP 3.1	DZP 0.028, NDZP 0.13	
10	Shot in head	Homicide	BUP 10, NBUP 8.3, BUP/NBUP 1.2	AMP 0.71, DZP 0.13, NDZP 0.16, oxazepam* <0.01, temazepam < 0.02, THC-COOH 0.011	AMP

LAI long-acting injectable, BUP buprenorphine, NBUP norbuprenorphine, AMP amphetamine, BZD benzodiazepine, EtOH ethanol, THC tetrahydrocannabinol, THC-COOH carboxy-THC, 11-OH-THC hydroxy-THC, PRG pregabalin, CLO clonazepam, DZP diazepam, NDZP nordazepam

substances marked with * were medications for which the patient had a prescription

^a)^ those post-mortem findings that refer to concomitant substances that were found in post-mortem toxicology but not mentioned at the OAT patient records as problematic substances for each patient

**Fig. 2:**
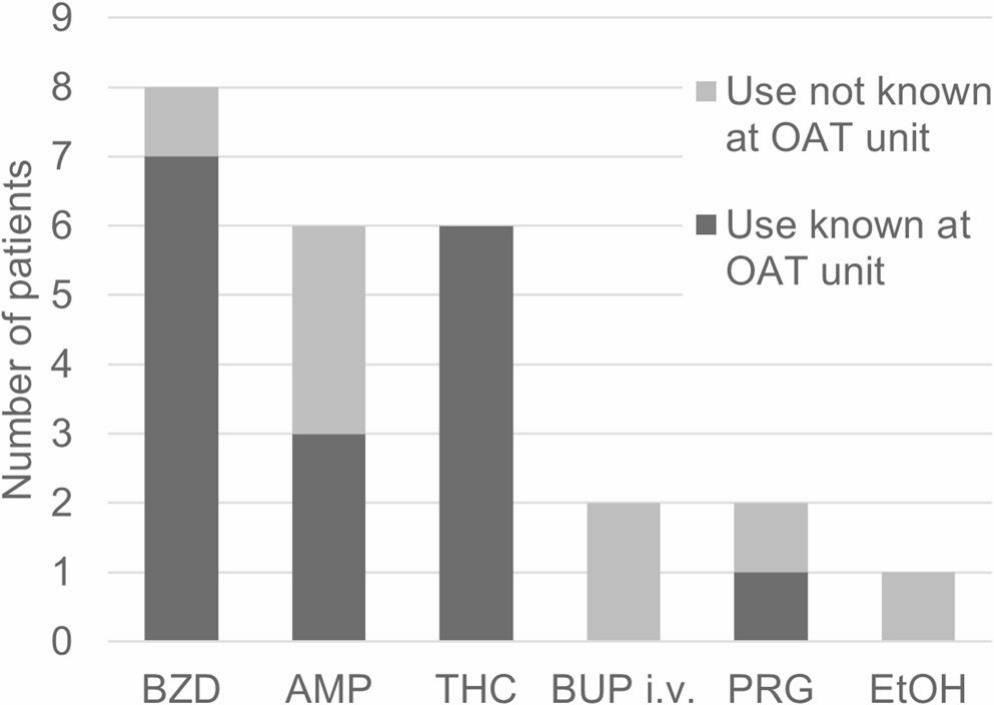
Concomitant substance use among fatalities with long-acting injectable buprenorphine treatment, classified by whether patient’s substance use was known at the opioid agonist treatment (OAT) unit or not. Data source: OAT patient records compared with findings in post-mortem toxicology BUP buprenorphine, BZD benzodiazepines, AMP amphetamine, THC tetrahydrocannabinol, i.v. intravenous, PRG pregabalin, EtOH ethanol

According to patient records, only one patient (patient #6) had no marked concomitant substance use during the treatment, and two patients had no PM findings of concomitant substances (Table 3). Four patients had PM findings of additional substances not classified as their regular substance use, three of them amphetamine. Eight patients had PM benzodiazepine findings. Four of them had prescriptions for oxazepam, but all of them also had unprescribed benzodiazepine findings. Gabapentinoids were present in two cases, but the only patient with prescribed gabapentin was additionally positive for pregabalin. One patient had known on-top tramadol use, but no other patient had known concomitant opioid use during OAT. Figure 2 summarizes concomitant substance use.

The median PM BUP concentration was 8.5 µg/l, and NBUP concentration 6.6 µg/l. Patients #4 and #9 had unexpectedly high BUP concentrations (Table 3), even though patient #4 had received his weekly injection six days before death, and patient #9 had received his monthly injection 22 days before. Blood BUP/NBUP ratios were also high, greater than 3. The high BUP concentrations likely indicate additional illegal buprenorphine use. No indication of known concomitant buprenorphine use was found in their patient records. No patients had PM naloxone findings, which would have indicated recent BUP-NAL abuse diverted from other OAT patients.

Findings of PM substances or known or unknown additional substance use did not appear to be connected to the compliance or non-compliance of OAT. According to the patient records, none of those with missed OAT visits or acute intoxications received extra psychosocial support or active referral to a withdrawal unit.

## Discussion

To our knowledge, this is the first study presenting fatalities associated with LAI BUP despite previous studies of the characteristics of buprenorphine deaths [13]. LAI BUP includes several benefits for OAT patients, OAT units, and society [3, 4, 6]. In 2020, in Finland, over half of the deceased BUP OAT patients received LAI BUP.

OAT in Finland is regulated by the Decree of the Ministry of Social Affairs and Health on the detoxification and substitution treatment of opioid addicts with certain medicinal products [14]. According to the legislation, OAT must be based on a treatment plan that specifies, besides pharmacotherapy, the objective of the patient’s treatment, other medical and psychosocial treatment of the patient, rehabilitation, and treatment follow-up [14]. According to Finnish treatment recommendations, OAT should always include individually planned psychosocial treatment [15]. A study that involved interviewing LAI BUP patients reported that it is essential to offer regular contact and additional forms of non-medical support to allow patients the best opportunity to succeed [16]. Despite the inevitable need for psychosocial treatment and rehabilitation, the results of our study demonstrate that LAI BUP treatment mainly consisted of delivering medication, while psychosocial treatment was lacking. Even patients who had recently started OAT received only minimal psychosocial support.

LAI BUP was initially recommended for compliant patients wishing to avoid daily visits to OAT units [3, 4]. Most Finnish OAT patients have concomitant use of ethanol, illegal drugs, or prescribable medications [9, 17]. In our study, nine out of ten patients had regular concomitant substance use, four had been in OAT less than six months before death, and three patients were classified as non-compliant with OAT. When OAT medication is delivered daily at the OAT unit, personnel meet the patients regularly and assess their need for extra support or withdrawal of concomitant substances. The three non-compliant patients in our data had shown signs of non-compliance with OAT, such as missed OAT visits and clinical signs of intoxication, but received no extra support. In OAT with LAI BUP, it might be even more essential to monitor patients carefully to recognize the need for additional support because patients visit OAT units less frequently than in BUP-NAL or methadone treatment. It seems that more actions could have been taken to support the LAI BUP patients’ treatment adherence before they died.

Concomitant substance use is a persistent concern among Finnish OAT patients [18]. In the United Kingdom, six months after initiation of LAI BUP, three out of 12 patients had used morphine or heroin, based on either self-reporting or urine drug screens, two had used cocaine, and none had used benzodiazepine or amphetamine [19]. According to the patient records in our study, one patient had known concomitant tramadol use, but none had known concomitant buprenorphine use. The PM toxicology results, however, suggested concomitant buprenorphine use by two additional individuals in this study. Previously reported average plasma BUP concentrations in intravenous BUP use, sublingual BUP OAT, and LAI BUP aligned with each other [20]. They were lower than the average PM whole-blood concentration in our study. For example, with a 128 mg LAI BUP dose, the maximum BUP concentration was reported to be 6.59 µg/l and the trough concentration 0.93 µg/l [20], whereas our patient #9 had PM BUP concentration of 21 µg/l 22 days after the injection. Non-prescribed benzodiazepines were detected in the patients. The potentially dangerous combination of benzodiazepines and buprenorphine is well documented [21]. Benzodiazepine prescriptions have been associated with an increased risk of fatal and non-fatal opioid overdose and all-cause mortality [22]. Co-prescription of benzodiazepines, z-drugs (zopiclone and zolpidem), and gabapentinoids has been associated with increased mortality among opioid-dependent individuals [23].

More deaths occurred among patients receiving LAI BUP than sublingual BUP-NAL, although BUP-NAL was the most common OAT medication at the time of this study. However, as the number of cases was relatively small, it is difficult to draw further conclusions from this disproportion. Only 8% of buprenorphine deaths in Finland occurred among OAT patients [9], but it remains unknown whether LAI BUP possesses a greater risk of OAT death. Future research is essential in order to reveal risk factors in LAI BUP treatment and fatalities.

The PM BUP and NBUP concentrations among patients in LAI BUP treatment, with the medians of 8.5 µg/l and 6.6 µg/l, respectively, were higher than previously reported in parenteral buprenorphine abuse. The PM median BUP concentrations in Finnish buprenorphine-related deaths were 1.2–1.4 µg/l [24] and 4 µg/l [9], in France 5.8 µg/l [25] and 2.2 µg/l [26], in Singapore 1.3 µg/l [27], in Sweden 0.4–2.7 ng/g [28], and in Australia 3.0 µg/l [13]. In our study, the blood BUP/NBUP ratio was greater than 1 in eight cases, and only three of them were determined to be buprenorphine poisonings. Previous studies have reported median BUP/NBUP ratios greater than 1 in buprenorphine poisonings, and < 1 in cases other than poisonings [24, 28]. In general, buprenorphine toxicity is difficult to assess by PM blood concentrations only, and consequently, urinary metabolite ratios have often been used to support diagnosis [29]. OAT medications and treatment practices are developing, which poses a challenge for forensic toxicology and the interpretation of toxicology results in the context of cause-of-death investigations.

### Strengths and weaknesses

The strength of our study was the population-based setting, which meant that our data most likely included all LAI BUP deaths in Finland. All unexpected deaths and possible poisoning deaths underwent a medico-legal cause-of-death investigation with toxicological analysis, and over 95% of the OAT units replied to our inquiry about OAT status and sent us patient records. Having all the patient records of the deceased OAT patients allowed us to thoroughly assess their OAT performance.

The small number of deaths in LAI BUP treatment was a limitation. No statistical comparisons between LAI BUP and other OAT medications or generalizations of mortality in LAI BUP treatment were possible.

## Conclusions

At the end of 2020, sublingual BUP-NAL was still the most common OAT medication, but more patients died during treatment with LAI BUP than with BUP-NAL. Based on laboratory findings and patient records, initially undiscovered concomitant buprenorphine use was likely in some cases and, in general, substantial concomitant substance use was common. The most common group of concomitant drugs was benzodiazepines. Among the deceased receiving LAI BUP, psychosocial treatment and support seemed minimal.
